# Clinician attitudes toward and use of electronic problem lists: a thematic analysis

**DOI:** 10.1186/1472-6947-11-36

**Published:** 2011-05-25

**Authors:** Adam Wright, Francine L Maloney, Joshua C Feblowitz

**Affiliations:** 1 Brigham and Women's Hospital, Boston, MA, USA; 2 Harvard Medical School, Boston, MA, USA; 3 Partners HealthCare, Boston, MA, USA

## Abstract

**Background:**

The clinical problem list is an important tool for clinical decision making, quality measurement and clinical decision support; however, problem lists are often incomplete and provider attitudes towards the problem list are poorly understood.

**Methods:**

An ethnographic study of healthcare providers conducted from April 2009 to January 2010 was carried out among academic and community outpatient medical practices in the Greater Boston area across a wide range of medical and surgical specialties. Attitudes towards the problem list were then analyzed using grounded theory methods.

**Results:**

Attitudes were variable, and dimensions of variations fit into nine themes: workflow, ownership and responsibility, relevance, uses, content, presentation, accuracy, alternatives, support/education and one cross-cutting theme of culture.

**Conclusions:**

Significant variation was observed in clinician attitudes towards and use of the electronic patient problem list. Clearer guidance and best practices for problem list utilization are needed.

## Background

Complete and accurate clinical documentation is a critical component of the care process. Medical records serve as an organizing structure for clinical decision making, a tool for communication to other providers, substantiation for billing, data for research and quality measurement and protection in the event of legal process. In 1968, Lawrence Weed, MD, published "Medical Records that Guide and Teach" which introduced the concept of the problem-oriented medical record (POMR) [1] and the ability to create and maintain a structured, coded problem list in a computer system. This advance radically altered medical record keeping, and also had important implications for how clinicians organized patient care and decision making processes.

Today, problem lists are widely used in both paper and electronic medical record systems. The ability to create a coded problem list is a requirement of the Certification Commission for Health Information Technology (CCHIT) for all certified electronic health record (EHR) systems [2] and problem documentation is an element of Joint Commission requirements [3]. Recently promulgated federal regulations for "meaningful use" of electronic health records mandate that physicians must document a coded problem (or a structured entry indicating that the patient has no problems) for at least 80% of their patients in order to qualify for substantial incentive payments.

Meaningful use is, of course, not the only or even primary reason why physicians would choose to use the problem list. First and foremost, problem lists are inherently useful clinically. An accurate problem list helps a physician to track a patient's status and progress, to avoid omissions in care and to organize clinical reasoning and documentation. The problem list is also critically useful when a clinician sees a new patient, giving him or her a "jumping off" point for the visit.

There is some evidence that patients who have accurate and complete problem lists receive better care than patients who do not. Hartung et al. conducted a study of patients whose left ventricular ejection fraction was below 40% (diagnostic of systolic heart failure). In this study, patients with heart failure on their problem lists were more likely to receive evidence-based care for their heart failure than patients who did not: 92.2% received an ACE inhibitor or ARB compared to 76.6% who did not have heart failure on their problem list, and similar patterns held for digoxin (61.1% vs. 36.7%) and spironolactone (26.7% vs. 13.3%) [4].

The problem list is also used for a variety of ancillary functions. For example, electronic problem lists can be valuable for generating diagnosis-specific registries [5]. Quality measurement programs (including "meaningful use" guidelines [6]) may use the problem list to define the denominator for measures, and researchers often identify study cohorts based on their documented problems. In addition, electronic clinical decision support systems [7-10] often depend on accurate clinical problem lists [11].

Despite its importance, the problem list is often incomplete. At our institution, we found that only 59% of patients with CAD have it documented on their problem list, with 62% documented for diabetes and 51% for hypertension. Similar results were reported by Szeto et al., who found 49% documentation for CAD, 42% for benign prostate hypertrophy and 81% for diabetes at a Veterans Affairs health center [12].

The purpose of this study was to learn why problem lists are so problematic. We chose qualitative techniques because they excel at answering "why" questions, can help us understand problem list utilization from the user's perspective, and can identify contextual and cultural factors that might affect attitudes and utilization [13-16]. In order to better understand clinician attitudes toward, and use of, electronic problem lists, we observed and interviewed a variety of clinicians. To assure the trustworthiness of our results, we triangulated using different methods, multiple researchers and sites, and types of subjects.

## Methods

### Setting

The study was conducted across the Partners HealthCare system, which includes the Brigham and Women's Hospital (Boston, MA), Massachusetts General Hospital (Boston, MA), Faulkner Hospital (Boston, MA), Newton-Wellesley Hospital (Newton, MA), North Shore Medical Center (Salem, MA) and several other smaller community and specialty hospitals. Partners also has a large community practice network called Partners Community HealthCare Incorporated (PCHI). PCHI practices are independently operated and generally small, with a mix of primary care, specialty and multi-specialty practices.

**Figure 1:**
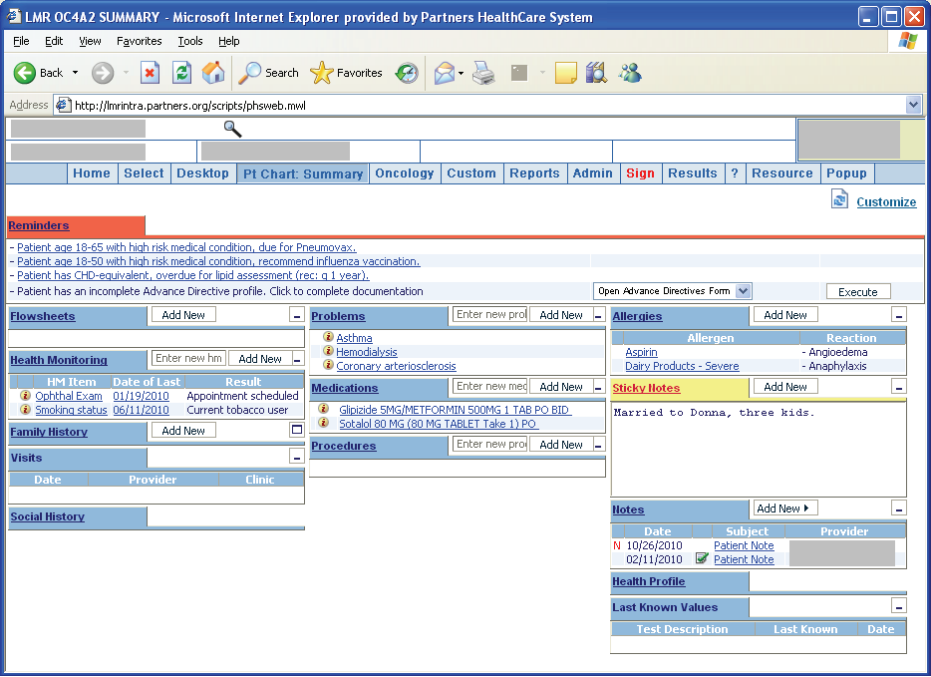
**The problem list as displayed in the LMR (top center)**.

All Partners clinicians are required to use an electronic health record (EHR) - most use the Longitudinal Medical Record system (LMR), a self-developed, CCHIT-certified EHR, but a variety of other systems are also in use (particularly the GE Centricity Physician Office EMR). The LMR contains an electronic patient problem list tool that allows providers to document coded and uncoded problem entries along with supporting detail. The problem list appears on the summary screen of the LMR (**Figure **1). Problem list use is not required and providers receive no specific guidance on use other than basic technical training.

### Sample

We received IRB approval from the Partners HealthCare Human Subjects Committee. We used a purposive sampling methodology for our study. We began with a list of all medical specialties represented across the Partners network, and selected a representative set of potential practices providing a broad spectrum of medical and surgical specialties, practice settings (hospital and community based, large and small, high and low socioeconomic patient status mix, rural and urban, etc.) and healthcare provider types (physicians, nurses, NPs, PAs, other allied health professionals and complementary and alternative healthcare providers).

We contacted clinicians and practice managers by phone and email, providing each with an information sheet describing our project. When clinicians agreed to participate, we scheduled in-person observations with them. We also purposively sought out particular types of clinicians, asking our subjects whether they could suggest others in their practice to observe. For example, we asked clinicians to suggest potential subjects who were high, typical and low users of the problem list. Occasionally, we would also add additional subjects in real-time if we saw a clinician during our observation who was willing to let us observe him or her on the spot. Data collection continued until a diverse sample of clinicians had been observed and saturation achieved.

### Field Work

Once clinicians agreed to participate, we scheduled observations at their site. Where possible we observed them for an entire session or shift (most typically a four-hour session, but sometimes an eight-hour shift or longer). Before the session, each clinician was provided with an IRB-approved information sheet and briefed on the study process by the investigators. We obtained verbal consent from our subjects. Where possible, we observed the clinician's entire use of the EHR and problem list, from pre-visit preparation to the actual clinical encounter (when the patient verbally agreed) to documentation. A two person team (AW and FM) conducted each observation.

In addition to observations, we also interviewed the subjects during down times in the clinic day, as well as at the end of our observations. These were short semi-structured interviews to explore problem list issues and also gave an opportunity for questions about our observations of the clinicians' behaviors.

We took extensive field notes throughout the observations and interviews, and occasionally recorded parts of interviews. These notes formed the basis for our analysis. Our field methods were based on the participant observation [17] and ethnographic interview [18] methods of Spradley, and were also influenced by the Rapid Assessment Process methods of Beebe [19], as adapted for assessment of clinical information systems by McMullen [20]. We chose these methods because they allow outsiders to relatively rapidly assess culture surrounding a focused area (in our case, the problem list) and to determine common and recurring themes.

### Debriefing

After we completed each observation, we met to debrief and review our notes. During each debriefing, we discussed what we had observed, and elicited a set of observations or quotations from each subject, such as "our leadership encourages us to maintain good problem lists" or "the problem list is not complete, therefore I don't rely on it".

### Analysis

We conducted a complete analysis of our field notes observations using a grounded theory approach [21]. We used an iterative card sort method to classify our observations into themes. Each of the two observation team members (AW and FM) initially sorted a representative sample of cards into sets, and then met to drive towards consensus to develop a coding scheme. We then did an independent card sort activity categorizing the remaining cards. After four rounds of iterative sorting, the classifications and themes converged, and we re-reviewed our field notes and observation catalog to identify patterns within the themes, and to review classes of providers exhibiting various aspects of those themes. We also did member checking, reviewing our themes with a subset of our subjects to ensure we had accurately captured meaning.

## Results

### Study Participants

We observed a total of 63 clinicians. Of these, there were 35 attending physicians, 2 fellows, 5 residents, 2 interns, 3 physician assistants, 4 nurse practitioners, 1 nurse anesthetist, 2 nurse midwives, 4 nurses, 2 social workers, 1 pharmacist, 1 massage therapist and 1 acupuncturist. Our sample included a wide variety of primary care clinicians (primary care, family medicine, pediatrics, geriatrics), surgical specialists (general surgery, otolaryngology, obstetrics and gynecology, anesthesiology) and non-surgical specialists (cardiology, medical oncology, endocrinology, infectious disease, emergency medicine, psychiatry).

### Observations

The observations were carried out over a nine month period from April 2009 to January 2010. We spent 264 hours observing and interviewing clinicians. Our observations ranged in length from 15 minutes to 8.5 hours. Most observations were initially scheduled for 4 or 8 hours but actual length of each session varied based on provider schedules. In addition, some shorter sessions were conducted spontaneously in the field as described in the Methods section. In one case, observation was stopped at a subject's request because the subject found it distracting. The mean observation time across all providers was 153 minutes.

### Findings

Across the 63 clinicians we observed, usage of and attitudes toward the problem list varied greatly. Some physicians were punctilious in their use of the list, constantly updating and refining entries in a manner consistent with Dr. Weed's model of the "problem-oriented medical record." Other physicians viewed themselves only as consumers of the problem list - reviewing it from time to time but never modifying or adding. Still others disregarded it entirely - neither viewing nor modifying the list (and in some cases saying they had never even heard of it).

### Themes

We identified a total of nine core themes and one cross-cutting theme to help categorize and explain our findings. These themes describe the full spectrum of problem list utilization behaviors observed (or elicited through interviews) in our sample. Attitudes were variable, and dimensions of variations fit into nine themes: workflow, ownership and responsibility, relevance, uses, content, presentation, accuracy, alternatives, support/education and one cross-cutting theme of culture (**Table **1). The results of our analysis are described in full below and include findings derived from both direct observation and clinician interviews. With each quote from an interview or observation session, the specialty of the associated clinician is provided; this is meant only to provide information on the source of the quote and does not to imply that a certain opinion was associated with a given specialty.

**Table 1:** Summary of themes

Theme	Aspects and Interconnections
Workflow	• Aspects
	○ Points in the clinical encounter where providers use the problem list
	○ Usability issues
	○ Delegation of problem list use
	• Interconnections
	○ Delegation often depended on attitudes towards ownership and responsibility
	○ Different workflows appeared depending on uses

Ownership and Responsibility	• Aspects
	○ Issues regarding what providers are responsible for maintaining the problem list, and which problems each provider is responsible for
	• Interconnections
	○ Providers felt more ownership when they saw relevance to their practice

Relevance	• Aspects
	○ The extent to which providers viewed the problem list as relevant to their practice
	○ Includes both intrinsic and extrinsic relevance (including authority)
	• Interconnections
	○ Relevance drives use and sense of ownership/responsibility

Uses	• Aspects
	○ All reported uses of the problem list by providers
	○ Included both adding problems and referring to problems, as well as non-clinical uses (billing, etc.)
	• Interconnections
	○ Perceived uses drive relevance
	○ Different uses often require different workflow

Content	• Aspects
	○ Concepts relating to provider opinions on appropriate (and inappropriate) types of problem list content
	• Interconnections
	○ Content relates to relevance, as providers are most interested in adding content they perceive to be relevant
	○ Provider attitudes towards ownership and responsibility affect their willingness to modify problem list content (e.g. to discontinue a problem added by another provider that they consider irrelevant or incorrect)

Presentation	• Aspects
	○ Observations related to actual and ideal representation of information in the problem list tool
	• Interconnections
	○ Different workflows and uses may have different optimal presentations

Accuracy	• Aspects
	○ Observations and opinions relating to the general accuracy, completeness and currency of patient problem lists
	• Interconnections
	○ Perceptions of accuracy affect uses and intention to use

Alternatives	• Aspects
	○ Any other mechanism of documenting problem list content other than the formal structured problem list
	• Interconnections
	○ Perception that alternatives are superior affects use and relevance attitudes

Support/Education	• Aspects
	○ Observations related to education and technical training on problem list use and ongoing support
	• Interconnections
	○ Support/education affect perception of uses and relevance
	○ Issues with workflow relate to sub-optimal support/education

Culture	• Aspects
	○ Local, institutional and professional culture around problem list use
	• Interconnections
	○ Cross-cutting theme influencing all other themes

#### Workflow

Clinicians integrated the problem list into their workflow in a variety of ways. Many users would review the list at the beginning of the encounter - in some cases before going in to see the patient, but in other cases in the exam room with the patient present. Some of the frequent users of the problem list used it to guide the entire encounter. During their interview with the patient, they would work through the problem list, asking questions and updating the record problem by problem, and adding problems in real time as new concerns were identified. Other providers held their problem list edits to the end of the encounter (or even to the end of the day or, less commonly, the end of the week when they finished the note for that visit).

In some cases, especially among surgeons, providers would delegate the documentation of problems to other providers, such as medical assistants or physician assistants. In other cases, providers simply did not use the problem list at all, preferring instead to document concerns in dictated notes (see the Alternatives theme) or felt that problem lists were not relevant to their type of care at all (see the Relevance theme).

Within the workflow theme, a series of usability issues also arose. Partners HealthCare uses a coded problem list with a dictionary lookup (and the option to use free-text entries). Providers were often disappointed that the problem list feature could not identify synonyms they might use or misspelled problem concepts. Some providers also felt that adding problems was too time consuming, saying "the summation of clicks is the real problem" (family practice), "it would add 3 hours to my day, which I'm really not interested in" (otolaryngology) or "I would do it if I had the time" (infectious disease).

Some clinicians proposed alternative methods, such as kiosk-based entry of problems by patients, or automated inference of problems. One endocrinologist commented that "to have the doctor entering that stuff seems so retro".

#### Ownership and Responsibility

One important related theme is the idea of ownership of or responsibility for the problem list. Many primary care providers felt responsibility for maintaining the problem list, though they also thought that specialists shared responsibility for maintaining the list. There was some disagreement among PCPs about whether specialists should limit their use of the problem list to their own specialty, or whether they should document comprehensively: "it's patient based, not me based" (primary care), "it [having specialists add problems related to their specialty] would make things more accurate, more timely" (primary care), "it's not realistic to have one person in charge" (infectious disease). However, many specialists felt that the problem list was the responsibility of the PCP, and that specialists had little (or no) responsibility for recording problems outside or even inside their specialty: "it's the PCP domain" (psychiatry).

The ownership theme also extended to modification of the problem list. Many providers said that they would be uncomfortable modifying or discontinuing problems added by another provider: "I would never mess with the problem list or medication list" (primary care). Receptiveness towards being on the receiving end of such modifications varied - many providers said they would be fine with changes; however, other providers were more reluctant: "I feel like it's my baby I created for my patients" and "I would be so pissed if they [specialists] deleted" (primary care).

#### Relevance

Related to the theme of ownership and responsibility was relevance. Many clinicians (especially primary care providers) felt that the problem list was relevant to the care they provided, but specialist opinions were mixed. The more enthusiastic providers said that "the problem list is the bedrock of medicine" (primary care) or that "we live by the problem list" (obstetrics). Many specialists also felt that the problem list was relevant, but some disagreed: "[I'm] just thinking bones" (orthopedic surgery) or "I think problem lists are updated every 6 months by anal residents in [the internal medicine clinic]" (general surgery).

In addition, some clinicians responsible for issues outside of direct medical care did not see the problem list as relevant to their roles. Some social workers reported that they do not diagnose problems, but instead identify concerns that wouldn't generally belong on the problem list. Complementary and alternative providers reported making some use of the problem list "to get a picture of what's going on with the patient, what they've been through and what I can do to help them." (massage therapy) but reported very rarely making modifications to the problem list (though they do write notes).

In addition to direct care, some clinicians were motivated to add problems because they are used in quality measurement and research: "someone's using this data for research, you don't want to use uncoded problems" (geriatrics). Others reported that, even if they didn't find the problem list personally relevant, they were motivated by a pay-for-performance target applicable in certain Partners clinics, by enhanced billing, by peer pressure or by their leadership.

#### Uses

A large number of observed and interviewed providers reported that they thought the problem list was useful. Even some non-users of the problem list agreed that, in the ideal scenario, the problem list could benefit patient care. Many providers reported that the problem list was especially valuable when caring for patients not previously known to the provider (either new patients transferring in, or in coverage scenarios), and direct clinical use was the most commonly reported use for the problem list.

Some providers also pointed out that the length of the problem list (apart from its content) was also an important indicator of a patient's overall health and disease burden. One provider also indicated that a very long problem list in conjunction with a very short medication list is a "red flag" for somatization.

Clinical decision support was also reported as an important driver for problem list utilization. Several providers indicated that they would add problems where they knew they would receive useful reminders (e.g. for diabetes). The LMR also has a "KnowledgeLink" function, allowing users to find reference information for problems, and some users reported this as an additional incentive to add problems. Some users also reported, anecdotally, that they had observed others who occasionally intentionally created uncoded, misspelled problem list entries (e.g. "diabetees") to suppress unwanted reminders.

Certain non-clinical uses of the problem list were also identified. Pharmacists and nurses reported using the problem list to find indications for prior authorization requirements of drugs. Many providers also use the problem list in billing, though some providers felt that this use did not always lead to clinically optimal problem lists, "I think others are padding the problem list for billing purposes" (infectious disease). Finally, several providers indicated that they thought the problem list was important in quality measurement and research, and so they added problems to support these functions.

#### Content

One significant area of disagreement about the problem list related to its content. There was widespread agreement that active, ongoing, chronic problems belong on the problem list. However, there was considerable disagreement about other types of data. Some providers included resolved problems, acute but likely self-limiting problems, family history, surgical history, symptoms without definite diagnosis (such as chest pain), medical devices, social issues, demographics.

In many cases, providers reported significant nuances in their attitudes towards these data types. For example, many providers thought that "status post myocardial infarction" belonged on the problem list, despite its latent or historical nature, but opinions on entries like "history of urinary tract infections" were much more mixed. The documentation of acute processes, like otitis media and cough, was also controversial - many providers favored documenting them and then inactivating them once resolved, others felt that such problems should only be elevated to problem list entries if recurrent. Some providers also added additional entries like "narcotics contract," and "Jehovah's Witness" to the problem list, though others emphatically believed these to not be problems. This behavior seems to represent a workaround due to a lack of other prominent places to track this information in the information system.

The actions users took in response to subjectively inappropriate existing content varied - many providers complained that they found problem lists to be filled with "junk" but wouldn't want to offend another provider by removing it (see also the Ownership and Responsibility theme), while others aggressively pared details they found irrelevant.

One significant facet of the content theme revolved around coded and uncoded problem list entries. The LMR provides a large dictionary of problem terms mapped to SNOMED, but users have the option to add uncoded terms (which cannot drive decision support) as needed. Many users reported that they did not know the difference between these two problem types (or even that there were different types available) despite an alert shown each time an uncoded entry is added. Some users who were aware of the difference reported a great desire to use coded terms "I bend over backwards to find a coded problem" (primary care), though this desire was often counterbalanced by some concern about the granularity of the problem terminology. For example, the LMR problem list dictionary contains "thyroid cancer", but does not allow for further specification of the type (papillary, follicular, medullary, etc.). Some users expressed uncertainty as to whether it was better to put a coded but more general term on the list, or an uncoded but more specific term. Some users reported that they had requested the addition of terms to the dictionary, but many of these users felt that their requests were ignored and quit making further requests.

#### Presentation

The LMR provides little support for organizing the problem list - users can reorder the list and mark problems as inactive, but there is no ability to automatically sort or group problems. Many users commented that they would like to be able to group the problem list by a variety of criteria, including chronology, status (active or resolved), disease course (acute or chronic), certainty (established or provisional), organ system or importance. Other users wanted the ability to create a problem hierarchy or otherwise represent relationships between problems (e.g. to show that a patient's diabetic retinopathy is part of their diabetes, and that their diabetes is, in turn, linked to obesity).

Some providers also wanted specialty specific views, or the ability to only show problems that they had entered. Others disagreed with this (sometimes quite emphatically) declaring that it's "bad medicine" (oncology) to only see problems in your specialty, and that "people should not live in a silo of I'm only treating xyz" (anesthesiology).

#### Accuracy

Many subjects commented on the accuracy and reliability of the problem list. Some thought the problem list was generally complete: "I usually take it at face value" (primary care), a number of subjects repeatedly stated they could not rely entirely on the problem list because it is not complete or accurate: "If I could trust it, I would look at it", "I don't use the problem list anymore because no one updates it". Providers reported that false negatives (missing problems) were much more common than false positives (incorrect problems). Some providers reported that they had confidence in the problem lists for their own patients, and sometimes for patients cared for by other physicians in their practice, but had less confidence in problem lists maintained by others.

For many providers, the lack of reliability meant that they would still review (and possibly update) the problem list, but that they will augment the problem list with other sources of information such as medications, notes, recent hospital discharge summaries or interviewing the patient (see the Alternatives theme). However, others reported completely discontinuing their use of the problem list, relying entirely on alternatives. We noted a "tragedy of the commons" occurring in many practice settings - providers reported that, frustrated with their incompleteness, they had stopped updating patient problem lists - this disuse then contributed to the further decay of the problem list, causing other providers to also discontinue use.

In contrast to this cycle of disuse, we also found some settings of mutually reinforcing use. A particular midwifery practice made consistent use of the problem list. When asked if the problem list was reliable, one midwife indicated that she could rely on the problem list "because it's always accurate". Asked if she were certain that the problem list was always accurate, she replied "it has to be accurate - we rely on it."

#### Alternatives

Many clinicians reported using a variety of alternative approaches to determining a patient's problems in addition to (or instead of) the structured problem list. The most common was keeping a problem list in the past medical history section of their outpatient progress notes. Clinicians also reported reviewing the most recent discharge summary (often cited as a very reliable snapshot of a patient's medical issues at the time of discharge), using the medication list to infer problems (particularly for medicines with a single or narrow set of indications), reviewing billing diagnoses (when available), relying on their own memory of the patient, discussing the patient with other providers or querying the patient directly.

Clinicians gave a variety of rationales for the use of these alternatives. One common explanation was their lack of confidence in the accuracy of the problem list (see Accuracy theme). However, clinicians also reported some specific adaptations to their style of care or personal preferences. For example, some specialists preferred to keep a personal specialty-specific problem list in their note to avoid the "clutter" of the complete problem list. Others said that they preferred to hear the patient describe their medical history in their own words. Finally, some providers kept shadow paper problem lists, reporting that they felt they could better organize their thoughts on paper than in a computer system. These problem lists are maintained outside the patient's standard medical record and cannot be readily accessed by other providers, which may ultimately result in fragmentation of clinical information due to unavailability or unawareness of these additional records.

#### Support/Education

Many users reported that they had received little or no formal education or training on the use of the problem list. Some indicated that they had been informally trained on the problem list during medical education, but this was variable. Within obstetrics, for example, one fellow reported being taught during medical education and residency to thoroughly document problems - another, who studied elsewhere, reported "I wasn't taught that way", and didn't begin using the problem list until she joined Partners.

Several subjects had received training on the LMR which included a short section on adding and modifying the problem list, but reported that this training was purely technical in nature, teaching them the mechanics of the problem list function in the LMR, but not providing any information on content, responsibility or effective use.

Most clinicians reported that requests for additions to the problem list dictionary were generally ignored, "When there's a problem or bug they work really fast and are really effective. When there's a new feature request it never gets done." (oncology), "I quit making suggestions because they never do them" (obstetrics), though some clinicians did report receiving occasional responses. We queried the Partners Knowledge Management group, which reported that there is a general hold on adding new problems that might impact decision support until after the Partners problem dictionary is fully migrated to SNOMED.

#### Culture

The final theme we identified was a cross-cutting theme of problem list culture. Much of the variation in attitudes towards problem list usage was attributable to various prior themes we reported; however, we noted that there appeared to be a slightly more amorphous notion of culture within practices - some practices and clinicians, otherwise similar, used the problem list more or less than others. This culture seems to be derived from a variety of sources - we observed cultures tied to medical specialties, particular institutions, particular clinics within institutions and particular types of healthcare providers within clinics.

Problem list culture appears to be a complex and multifactorial phenomenon. In many cases, it is driven by formal leadership - some physicians reported that they used the problem list principally because the clinical leadership of their clinic set an expectation that everyone would use the problem list, and followed this with periodic chart audits and feedback to clinicians. This leadership did not always require formal authority - in some clinics, there was a single "champion" without formal authority who encouraged his or her fellow clinicians to maintain accurate problem lists. A culture of problem list utilization can also be driven by a sort of clinical "citizenship". In many settings, providers reported that they often took care of each others' patients, and that they depended on an accurate problem list when providing coverage, so they prioritized keeping their own problem lists updated for the benefit of their fellow providers.

Qualities inherent to a specialty or practice setting, as well as a specialty's larger culture may also drive the formation of a problem list culture. Specialties providing longitudinal care to patients, such as primary care and oncology, had strong cultures of problem list utilization, while episodic care settings, such as surgery, did not have a strong culture around problem list use. However, the obstetrics practices we observed were consistent users of the problem list - though they do provide episodic care (frequently of a surgical nature) their episodes are longer than many other surgeons', they provide frequent, ongoing and sometimes high-intensity care to their patients, and they have a strong cultural tradition of cross-coverage and mutual dependence (when an obstetrician encounters a patient in labor he or she expects to find accurate and complete documentation of the patient's prenatal course written by the extended obstetrical care team).

## Discussion

### Recommendations

From our study, it became clear that there was tremendous variation in provider attitudes towards electronic problem list use and, in many cases, considerable frustration. Almost all clinicians (even non-users) agreed that the problem list was important and potentially useful, but many also felt that its full potential was not being realized.

One especially important phenomenon that we detected was a wide range of opinions on how the problem list should be used. This manifested itself particularly in the Ownership and Responsibility, Accuracy and Content themes. Providers expressed widely divergent views on who should be responsible for the problem list, the degree to which problem lists were reliable and up-to-date and the appropriate kinds of information to document in the problem list.

This variability suggests that more research, and perhaps policy setting, is needed to ascertain best practices for problem list usage and ultimately, perhaps, to establish guidelines or policies. None of the hospitals or clinics we studied had a formal policy on who is responsible for maintaining the problem list or what clinical conditions should belong on the problem list. Some clinicians reported an implicit or explicit expectation that they updated the problem list, but none had received specific guidance or training on effective problem list utilization, and several reported uncertainty about ownership of and responsibility for the problem list. The lack of a formal policy may have contributed to the wide variety of problem list use behaviors that were observed in this study.

A consistent policy on problem list use within and across institutions would likely be beneficial for increasing the value of this shared resource for all providers. We believe that clinicians and clinical professional organizations are optimally positioned to devise best practices (and perhaps model policies) and encourage them to do so. These findings also have implications for EHR developers who should collaborate with guideline developers to augment electronic problem list tools.

Developing consensus on optimal use of the problem list would have considerable benefits. Establishing wider agreement could make documentation more consistent, thus reducing inaccuracies and making patient problem information more readily accessible. This, in turn, could further increase use of this shared resource if providers find problem list content to be more consistently reliable and have a clearer understanding of expectations for its management.

Additionally, the federal meaningful use regulation (Stage 1) explicitly mentions the problem list, requiring that "more than 80 percent of all unique patients seen by the [eligible provider] have at least one entry or an indication that no problems are known for the patient recorded as structured data" [6]. This will likely result in an increase in use of electronic problem lists but does little to ensure their accuracy and consistent maintenance. The regulations (perhaps appropriately) do not specify what is or is not a problem, nor, in the setting of a shared record between specialists and primary care providers, who is responsible for maintaining the problem list. To meet this goal, as well as future Stage 2 and 3 benchmarks, more consistent problem list use will be needed.

Here we have identified major themes in provider attitudes and use of the problem list using qualitative methods. Future research should expand this analysis to other sites to test the generalizability of these findings and should incorporate quantitative analysis of provider problem list use. Formal guidelines should then be developed on the basis of these findings.

### Limitations

Our study has some important limitations. First, as is the case with any ethnographic study, our results are inherently influenced by the ethnographers themselves. We adhered to standard ethnographic methods to manage potential bias and to ensure that our data collection frame was open and wide, but it is possible that other observers might develop different conclusions.

Second, though our sample was large and diverse, it was limited to a single health system, and focused specifically on providers using an EHR, which limits the generalizability of our results. Given all providers used the same problem list tool, some of the issues reported, especially those around workflow, may be tied to the advantages and deficiencies of this particular system. It is likely that providers outside of our system, or non-users of an EHR might be systematically different. To guard against the first issue, we explicitly asked our subjects about their experiences before joining Partners (subjects came from a variety of community, academic, military and institutional practice settings) and integrated this information into our analysis. The second issue (focus on EHR users) was inherent to our study design. We would encourage other researchers to explore problem list utilization using similar techniques in other settings and among non-EHR users - such study would almost certainly add additional richness to our findings.

Third, and finally, our study may be subject to the Hawthorne effect (subject reactivity to observation). We did our best to guard against this by encouraging subjects to speak honestly and probing them about whether behaviors they exhibited were typical. We also used the ethnographic technique of triangulation: in addition to observations, we also used interviews, observed multiple subjects in most settings, and occasionally reviewed past records and problem list entries in real time with our subjects to ensure a complete and unbiased understanding of their problem list attitudes and behaviors.

## Conclusion

Clinicians do see the intrinsic value in accurate, up-to-date problem lists; however, real-world usage of problem lists is highly variable and often falls short of ideal. We identified important issues regarding reliability of the problem list, as well as lack of consensus (and even confusion) about ownership and responsibility, content and perceived relevance of the problem list. Resolution of these issues may enable more effective and efficient use of the problem list, potentially resulting in improved quality of care.

## Abbreviations

POMR: Problem-oriented medical record; CCHIT: Certification Commission for Health Information Technology; EHR: Electronic health record; ACE: Angiotensin-converting enzyme; ARB: Angiotensin receptor blocker; CAD: Coronary artery disease; PCHI: Partners Community HealthCare Incorporated; LMR: Longitudinal Medical Record; EMR: Electronic medical record; IRB: Institutional Review Board; PCP: Primary care provider; SNOMED: Systematized Nomenclature of Medicine

## Competing interests

The authors declare that they have no competing interests.

## Authors' contributions

AW conceived the study, participated in its design, observations, and analysis, and drafted the manuscript. FM coordinated the participants of the study, participated in observations and analysis, and helped with the draft of the manuscript. JF participated in editing and revision of the manuscript, as well as additional analysis of the qualitative data and linking of themes. All authors read and approved the final manuscript.

## Pre-publication history

The pre-publication history for this paper can be accessed here:

http://www.biomedcentral.com/1472-6947/11/36/prepub
